# TCox: Correlation-Based Regularization Applied to Colorectal Cancer Survival Data

**DOI:** 10.3390/biomedicines8110488

**Published:** 2020-11-10

**Authors:** Carolina Peixoto, Marta B. Lopes, Marta Martins, Luís Costa, Susana Vinga

**Affiliations:** 1 INESC-ID, Instituto Superior Técnico, Universidade de Lisboa, Rua Alves Redol 9, 1000-029 Lisboa, Portugal; anacpeixoto@tecnico.ulisboa.pt; 2 NOVA Laboratory for Computer Science and Informatics (NOVA LINCS), FCT, UNL, 2829-516 Caparica, Portugal; marta.lopes@fct.unl.pt; 3 Centro de Matemática e Aplicações (CMA), FCT, UNL, 2829-516 Caparica, Portugal; 4 Instituto de Medicina Molecular-João Lobo Antunes, Faculdade de Medicina, Universidade de Lisboa, Avenida Professor Egas Moniz, 1649-028 Lisboa, Portugal; marta.martins@medicina.ulisboa.pt (M.M.); lmcosta@medicina.ulisboa.pt (L.C.); 5 Oncology Division, Hospital de Santa Maria, Centro Hospitalar Lisboa Norte, 1649-028 Lisboa, Portugal

**Keywords:** regularized optimization, Cox regression, survival analysis, TCGA data, RNA-seq data

## Abstract

Colorectal cancer (CRC) is one of the leading causes of mortality and morbidity in the world. Being a heterogeneous disease, cancer therapy and prognosis represent a significant challenge to medical care. The molecular information improves the accuracy with which patients are classified and treated since similar pathologies may show different clinical outcomes and other responses to treatment. However, the high dimensionality of gene expression data makes the selection of novel genes a problematic task. We propose TCox, a novel penalization function for Cox models, which promotes the selection of genes that have distinct correlation patterns in normal vs. tumor tissues. We compare TCox to other regularized survival models, Elastic Net, HubCox, and OrphanCox. Gene expression and clinical data of CRC and normal (TCGA) patients are used for model evaluation. Each model is tested 100 times. Within a specific run, eighteen of the features selected by TCox are also selected by the other survival regression models tested, therefore undoubtedly being crucial players in the survival of colorectal cancer patients. Moreover, the TCox model exclusively selects genes able to categorize patients into significant risk groups. Our work demonstrates the ability of the proposed weighted regularizer TCox to disclose novel molecular drivers in CRC survival by accounting for correlation-based network information from both tumor and normal tissue. The results presented support the relevance of network information for biomarker identification in high-dimensional gene expression data and foster new directions for the development of network-based feature selection methods in precision oncology.

## 1. Introduction

Colorectal cancer (CRC) is one of the leading causes of mortality and morbidity in the world. It is the third most commonly occurring cancer in men and the second in women, accounting for approximately 1.8 million new cases in 2018 and 880,792 deaths worldwide [1].

The pathogenesis of CRC results from the accumulation of genetic and epigenetic alterations that lead to the transformation of normal glandular epithelial cells into invasive adenocarcinomas. The majorities of CRCs (75%) are sporadic in origin and occur in people without genetic predisposition or family history of CRC. The other cases are familial or related to inflammatory bowel diseases [2].

Several types of genomic instability have been described in CRCs and may facilitate the acquisition of multiple tumor-associated mutations such as chromosomal instability, which generates gene deletions and duplications and occurs in 70–85% of CRCs, and microsatellite instability, characterized by mutations at nucleotide repeat sequences and accounting for 15% of sporadic CRCs [3,4]. This genomic instability may lead to a higher inter-patient and intra-tumor heterogeneity, being a great challenge for both diagnosis and cancer therapy [5,6]. Thus, it is essential to understand the molecular basis of individual susceptibility to colorectal cancer and to determine factors that initiate tumor development, drive its progression, and determine its responsiveness or resistance to antitumor agents.

During the past few years, high-throughput functional genomics has made notable progress. The development of novel high-throughput sequencing techniques such as RNA sequencing (RNA-seq) provided new methods for mapping and quantifying transcriptomes [7]. Furthermore, RNA-seq allows the study of the gene expression profile of thousands of genes simultaneously, providing a better view of the genetic pathways, showing genes that may be highly correlated or redundant [8]. Moreover, this rising of genome sequencing technologies contributes to more precise medicine, where the molecular information improves the accuracy with which patients are classified and treated [9]. Indeed, molecular data are particularly important in cancer studies, where patients with similar pathologies may show different clinical outcomes and different responses to treatment [10].

However, the high dimensionality of gene expression data makes the selection of novel biomarkers a difficult task, since the number of individuals (*N*) is typically much smaller than the number of genes (*p* covariates). In fact, N≪p leads to a high-dimensional problem that may cause instability in the selected genes [11]. Thus, to lower the dimensionality of the data, feature selection via model regularization has been applied in classification and also Cox survival models in the context of precision oncology [10,12,13]. For instance, in Cox regression, this corresponds to adding a penalty term to the partial log-likelihood of the Cox model, which sets some variables’ coefficients to zero. The Elastic Net (EN) penalty [14] and its particular case of the Least absolute shrinkage and selection operator (Lasso) [15] are state-of-the-art strategies for regularization-based feature selection.

Extensions to the above penalties to account for network-based information have been proposed in the context of cancer genomics. Penalty terms based on centrality measures of the nodes (genes) in the network have been suggested, such as the degree, therefore penalizing the variables based on their role in the overall network [12,16], and also by promoting the smoothness of the parameters across adjacent nodes in the network [17]. Network-based regularizers built on the correlation between the variables in different groups have also been proposed [13,18]. The central premise is that biomolecular networks in different cancer or cell types exhibit distinct network-based correlation patterns that might be regarded as biomarkers for disease/cell typing, but also similarities whose relevance might be investigated in the definition of common therapies for distinct disease conditions. Correlation has long been used for feature selection in classification and regression problems [19], in high-dimensional benchmark datasets [20], for early diagnosis and cancer progression based on cancer and normal biomolecular networks [21], for multivariate differential coexpression analysis between two conditions based on the complete correlation structure between genes [22], and for weighted gene co-expression network analysis for the discovery of the relationship between networks/genes and phenotypes in cancer, e.g., disease stage and overall survival [23,24].

In this work, we propose TCox, a correlation-based regularizer for feature selection in Cox regression models applied to transcriptomic data. This regularizer considers the differences in correlation between genes’ networks in healthy and in cancer tissues, promoting the selection of genes with different correlation patterns in the two conditions. The key underlying hypothesis of TCoxis that a gene with distinct interactions in the normal and tumor groups, given by its correlation with the other genes in the network, might have a potential association with patient survival. This regularizer was applied to colorectal cancer RNA-seq data to identify key genes in the survival outcomes and putative therapy targets of cancer patients.

## 2. Materials and Methods

**Figure 1:**
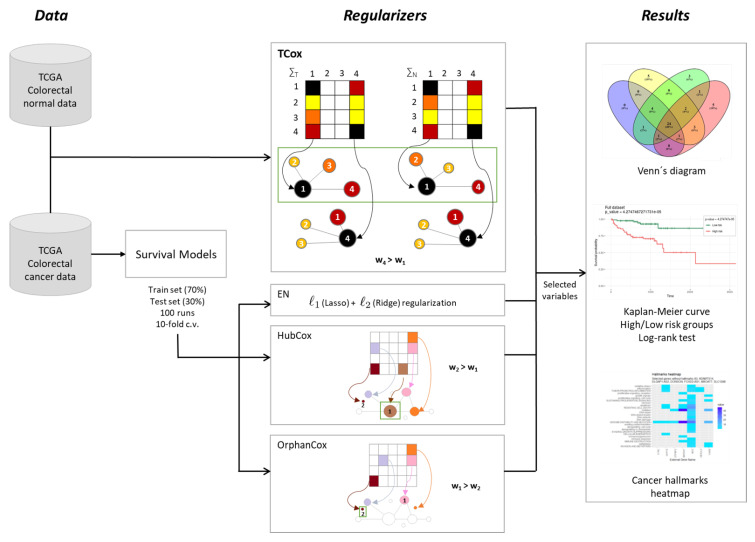
Methodological procedure for the identification of gene signatures in colorectal cancer data.

To disclose transcriptomic signatures in CRC, the model performances of survival models based on regularized Cox regression were evaluated over a range of different model parameters and data partitions. The analysis pipeline of this study is described in Figure 1.

### 2.1. Datasets

Transcriptomic and clinical data of colorectal cancer patients were obtained from The Cancer Genome Atlas (TCGA) through the Genomic Data Commons (GDC) data portal [25]. Colon Adenocarcinoma (COAD) and Rectum Adenocarcinoma (READ) RNA-seq Fragments Per Kilobase per Million (FPKM) data were imported using the RTCGAtoolbox R package [26]. The COAD transcriptomic dataset is comprised of 20,501 variables (genes) for a total of 328 samples (patients), 282 corresponding to primary solid tumor and 46 to normal tissue samples; the READ dataset has 20,501 variables for a total of 105 samples, 91 corresponding to primary solid tumor and 14 to normal tissue samples. Both datasets were merged and used for further analysis. Regarding clinical data, the colorectal cancer patient *status* (dead or alive) and *days to death* variables were selected for 595 samples. A total of 357 samples with both clinical and RNA-seq data were used for further analysis.

### 2.2. Survival Analysis

The analysis of the course of a disease in time is a crucial feature for cancer characterization, including prognosis and optimal therapies’ definition [27]. Survival analysis studies the time until an event of interest occurs (such as death) [28]. An inherent feature of survival times is that sometimes, the event of interest is not observed, either because the patient dropped out of the study or the study finished and the event did not occur during that time-frame, thus leading to censored survival times [27]. The Kaplan–Meier method allows the estimation of the population’s proportion that would survive given a particular length of time, under the same circumstances, using both complete and censored survival times [28]. The comparison of the survival curves of two groups is often performed using a formal non-parametric statistical test called the log-rank test [29]. To adjust for multiple variables or factors, the proportional hazards regression model was proposed [30] and is briefly described below.

#### 2.2.1. Cox Regression

The Cox regression model is a multiple regression model for the analysis of censored survival data. It is used to study the association between the features and the hazard function through [27]. The hazard function gives the instantaneous potential (per unit time) for the event of interest to occur, given that the individual has survived up to that time [31].

(1) $$h_{i}\left(t\right)=h_{0}\left(t\right)\exp\left(x_{i}^{T}\beta \right),$$

where $h_{i}\left(t\right)$ represents the hazard function of individual i=1,…,n, $h_{0}\left(t\right)$ represents the baseline hazard, $x_{i}={\left(x_{i1},x_{i2},\ldots ,x_{ip}\right)}^{T}$ are the measured covariates, and $\beta =(\beta_{1},\beta_{2},\ldots ,\beta_{p})$ are the regression coefficients.

The inference is made by maximizing the partial log-likelihood, given by:

(2) $$l\left(\beta \right)=\sum_{i=1}^{n}\delta_{i}\left(x_{i}^{T}\beta -\log\sum_{j\in R_{i}}\exp\left(x_{j}^{T}\beta \right)\right),$$

where $R_{i}=R\left(t_{i}\right)=\left\{j:t_{j}\geq t_{i}\right\}$ denotes the set of all individuals that are at risk at $t_{i}$, i.e., with a follow-up time greater than or equal to $t_{i}$, and $\delta_{i}$ indicates if the event was observed ($\delta_{i}=1$) or not ($\delta_{i}=0$) for patient *i*.

Model regularizers have been proposed to cope with the high-dimensional nature of modern datasets, such as gene expression data, comprising thousands of highly-correlated features. In Cox regression, a penalty term F(β) is added to the partial log-likelihood l(β) of the Cox model. In particular, the Elastic Net (EN) penalty, given by:

(3) $$F\left(\beta \right)=\lambda \left\{{\alpha ∥\beta ∥}_{1}+\left(1-\alpha \right){∥\beta ∥}_{2}^{2}\right\},$$

combines two different regularizers, the ridge penalty ($\ell_{2}$-norm regularization), which shrinks the coefficients and helps to reduce the model complexity, and the Lasso ($\ell_{1}$-norm regularization), which can lead the coefficients to zero, therefore performing feature selection [14]. The penalty is controlled by α and bridges the gap between Lasso (α=1) and ridge (α=0).

Network-based regularizers have also been proposed in the context of cancer genomics. The glmSparseNet package generalizes sparse regression models including a network-based regularizer when genes show a graph structure [12]. The models are built based on the glmnet [32] family of models, by including centrality measures of the network as penalty weights in the regularization term. The resulting network-based penalty is related to the weights attributed to each gene or node, either promoting highly connected genes (hub genes) or isolated genes (orphan genes) [12].

#### 2.2.2. TCox

To identify features (genes) that have distinct roles in cancer and normal tissue, we propose TCox. This new weighted regularizer promotes the selection of genes with distinct correlation patterns across tumor and normal tissue through Cox regression. TCox departs from a recently proposed method that also uses a correlation-based regularizer and exhibits promising results in identifying biomarkers [13]. The twiner is based on sparse logistic regression and enables the selection of gene signatures shared by two diseases in breast and prostate cancer. The correlation structure was also relevant to identify heterogeneity factors in glioblastoma [18]. Instead of trying to retrieve similar correlation patterns, TCox promotes genes that exhibit distinct relationships between two groups, thus highlighting potential differences in the corresponding sub-networks.

Given the tumor and normal datasets, TCox builds the correlation matrices, $\sum_{T}=\left[\sigma_{1}^{T},\sigma_{2}^{T},\ldots ,\sigma_{p}^{T}\right]$, and $\sum_{N}=\left[\sigma_{1}^{N},\sigma_{2}^{N},\ldots ,\sigma_{p}^{N}\right]$, respectively. Each column $\sigma_{j}$ corresponds to the correlation of gene *j* with the remaining ones. The dissimilarity measure of gene *j* between the two datasets can be defined as:

(4) $$d_{j}\left(T,N\right)=\arccos\frac{<\sigma_{j}^{T},\sigma_{j}^{N}>}{∥\sigma_{j}^{T}∥\;\cdot \;∥\sigma_{j}^{N}∥},j=1,\ldots ,p.$$

Two patterns are considered identical if the angle between the corresponding vectors is zero. In the context of this work, since we were looking for dissimilarities (tumor vs. normal), angles equal to zero were discarded. The goal is not to select genes that exhibit the same correlation pattern between tumor and normal tissues, but rather identify those that behave very differently in the two tissue types, i.e., being correlated in distinct ways.

The dissimilarity term is then normalized by their maximum value, as follows:

(5) $$w_{j}=\frac{d_{j}\left(T,N\right)}{\max_{k}d_{k}\left(T,N\right)},\;j,k=1,\ldots ,p.$$

The resulting w vector is then used as a weight factor in the EN regularizer, controlling how much the parameter λ affects each coefficient, as follows:

(6) $$F\left(\beta \right)=\lambda \left\{{\alpha ∥w\circ \beta ∥}_{1}+\left(1-\alpha \right){∥w\circ \beta ∥}_{2}^{2}\right\}.$$

where ∘ represents the Hadamard or entry-wise vector product, i.e., $w\circ \beta =w_{1}\beta_{1}+\ldots +w_{p}\beta_{p}$.

Genes with a larger dissimilarity between the two correlation matrices are less penalized in TCox, which does not hold in the present form of *w*. With the goal of favoring the selection of the most dissimilar genes across tumor and normal correlation data matrices, several transformations of *w* were considered and tested, namely 1−w, $1-w^{3}$, ${\left(1-w\right)}^{3}$, $\frac{1}{w}$, $\exp(-w^{3})$, and $\exp({\left(1-w\right)}^{3})$.

**Figure 2:**
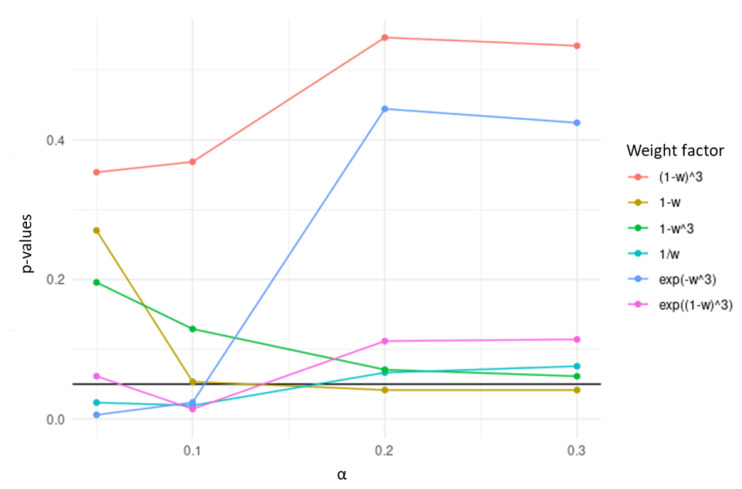
*p*-values obtained in the separation of high- and low-risk survival curves based on the genes selected by TCox models generated with transformations of *w* using colorectal RNA-seq data, tested over different α values.

Among the transformations tested using colorectal RNA-seq data, the $\frac{1}{w}$ transformation was chosen, since it yielded the lowest *p*-values in the separation of high- and low-risk survival curves, over the values of α evaluated (Figure 2). In the resulting penalty factor, for a certain gene in the network, the more different the correlation pattern across datasets is, the less penalized it will be in the regularization term of the Cox regression.

To evaluate the accuracy of TCox, we compared this approach with the above-mentioned survival methods, namely Cox regression based on the EN penalty, herein called EN, and HubCoxand OrphanCox models. TCox and Cox regression based on EN were built using the glmnet R package and the HubCox and OrphanCox models using the glmSparseNet package.

### 2.3. Model Evaluation and Comparison

Samples were randomly divided into a training set for model construction and a test set for model evaluation, comprising 70% and 30% of the data, respectively. Both subsets had the same proportion of censored samples.

The survival analysis was performed using four models: EN, HubCox, OrphanCox, and TCox. All models were estimated from 100 randomly generated runs with α=0.1 for both the training and the test sets. Among the 100 runs tested, only a few were statistically significant (Table 1), and none yielded significant results for the four methods simultaneously in the test set. The results presented hereafter were obtained using the run that showed statistically significant results for the test set in three models: TCox, HubCox, and EN. Afterwards, to analyze the level of sparsity of the models using the same partition obtained earlier, the α parameter was set between α=0.3 and α=0.05, which provides a feasible number of features to be further analyzed. To evaluate the performance of the models, the observations were split into two groups defined by the median of the fitted relative risks. This procedure allows performing the log-rank test via the Kaplan–Meier estimator and assessing if the two groups’ mortality is the same by evaluating the corresponding *p*-values. The selected variables using α=0.1 were compared between models and queried in the CHAT (Cancer Hallmarks Analytics Tool [33]) to assess the association between the selected genes and cancer hallmarks based on previous studies.

### 2.4. Availability of Data

All the implementations and R code described are freely available at https://github.com/sysbiomed/TCox, thus ensuring full reproducibility of the presented results. To perform all the analysis, we used the following R packages: to download TCGA data, we used RTCGAToolbox; regarding general preprocessing and visualization, we used dplyr [34], ggplot2 [35], and survminer [36]; for differential gene expression analysis, we used edgeR [37]; and for survival analysis and regularization, we used survival [38], glmnet [32], glmSparseNet [12], and biospear [39].

## 3. Results and Discussion

**Figure 3:**
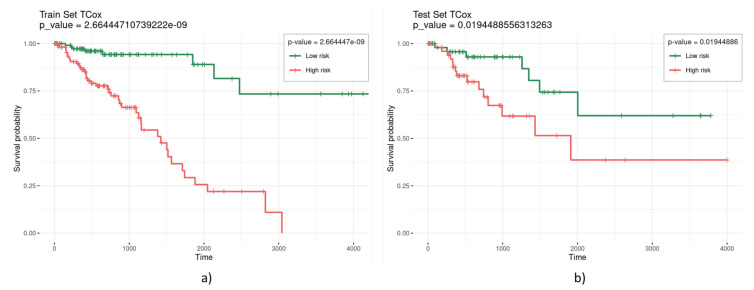
Kaplan–Meier curves obtained from the (**a**) training and (**b**) test sets, based on the variables selected by the TCox model with α=0.1.

TCox regression models were built based on the TCGA colorectal RNA-seq data from tumor and normal tissue samples to find a molecular signature comprising genes with a distinct correlation pattern in tumor and normal tissue networks. For biomarker and model evaluation, three different α were considered (0.3, 0.2, and 0.1) for the run chosen, thus selecting a different number of variables (Table 2). Most α values enabled the selection of a set of variables yielding significance (given by a *p*-value lower than 0.05) in the separation of the survival curves of high- and low-risk patients for the test set. Figure 3 illustrates a representative survival curve based on the variables selected by the TCox model in the training and test datasets, highlighting the significance of the selected gene set in the separation of the two risk groups.

The accuracy of the TCox survival model was compared against a Cox model with the EN penalty, HubCox, and OrphanCox survival models. Overall, in most runs, models were not able to significantly separate high- vs. low-risk groups (Table 1). Within the 100 runs tested using α=0.1, only a few runs were statistically significant in terms of the log-rank test using the estimated Cox parameters and median risks. The percentage of data partitions for which the models could not be estimated was 33% (TCox), 31% (EN), 43% (HubCox), and 32% (OrphanCox). Concerning the significant runs (*p*-value <0.05), the 4%, 3%, and 2% significant runs were obtained with EN, HubCox, and OrphanCox models, respectively, whereas TCox yielded 7% significant runs. These results may be an indication that the model performance is highly dependent on the data partition and might foster further research directions to cope with this limitation [40]. Besides these techniques, we also tested adaptive Lasso to evaluate other methods that are also based on sparsity and weighted regularization. However, the results were not statistically significant and, therefore, were not included.

Regarding the variables selected by the models, genes that were selected for at least 50% or 75% of the runs are listed in Table 3. One of the genes, *ELFN1*, was selected in at least 50% of the runs by the EN, HubCox, and TCox models. Interestingly, it was demonstrated that this gene enhanced both cell proliferation and migration in CRC [41].

**Figure 4:**
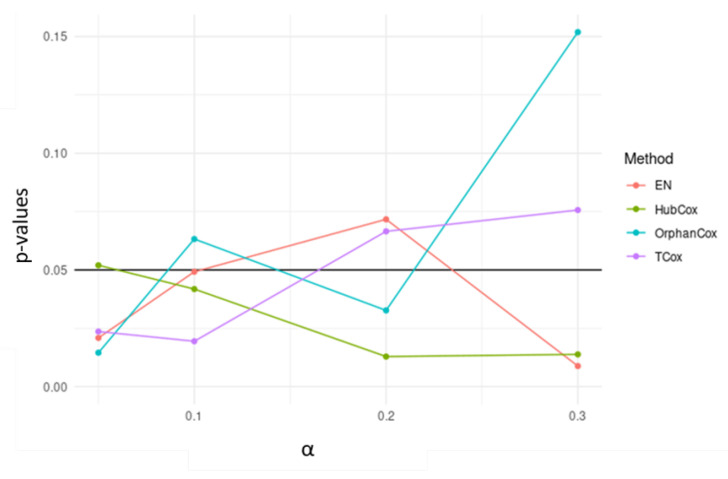
*p*-values obtained for survival models applied to the test sets, using different α-values.

**Figure 5:**
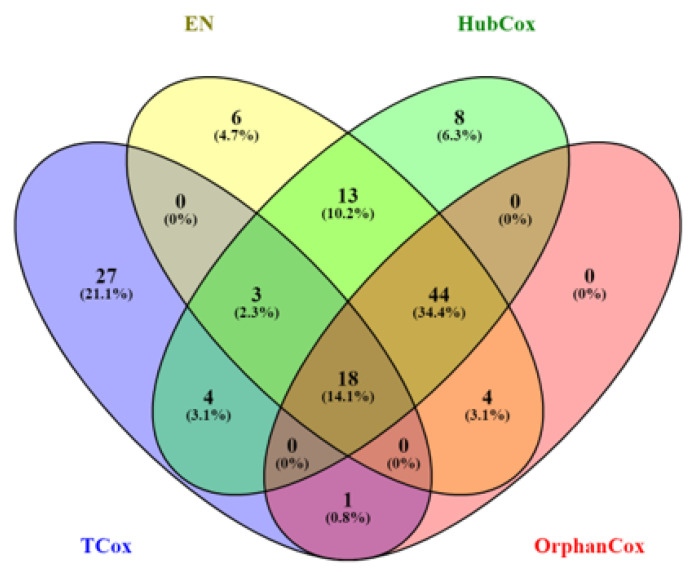
Venn diagram representing the number of genes selected by EN (yellow), HubCox (green), OrphanCox (red), and TCox (blue) using α=0.1.

Considering the results obtained for the representative run selected, TCox showed the lowest *p*-value for α=0.1 in the test set (Figure 4). When comparing the genes selected by the models tested using α=0.1 (an α-value that selected a reasonable number of genes to be further evaluated), some of the genes found, i.e., 18 genes, were selected by all four models (Figure 5).

Differential gene expression analysis using the edgeR package was performed to assess which genes were found to be up- or down-regulated in tumor tissue (Table 4).

**Figure 6:**
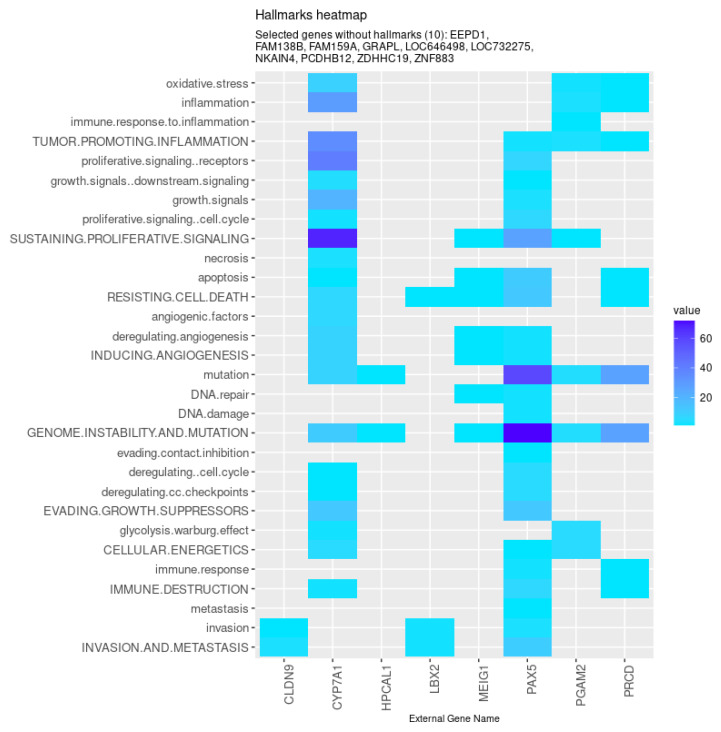
Genes selected by all models tested associated with the hallmarks of cancer, given by the CHAT. Value corresponds to the number of hits found in the literature, where light and dark blue correspond to a low and high number of hits, respectively.

Among those, eight genes were found to be associated with the hallmarks of cancer (Figure 6). Specifically, the models identified genes involved in metabolism (*CYP7A1* and *PGAM2*), tight junction formation (*CLDN9*), photoreceptor stability and transduction (*PRCD* and *HPCAL1*, respectively), genomic integrity (*MEIG*), and transcription regulation (*LBX2* and *PAX5*). Furthermore, besides some genes previously uncharacterized (such as *FAM159A*, *ZNF883*, and *LOC646498*), the models also selected non-coding RNA sequences (*LOC732275* and *FAM138B*) and protein-coding genes involved in cellular adhesion (*PCDHB12*) and DNA double-strand break repair (*EEPD1*), processes highly relevant in the context of cancer.

**Figure 7:**
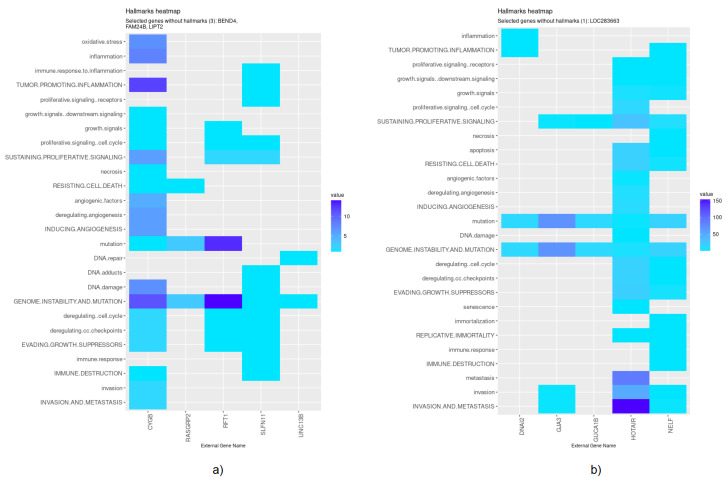
Genes selected by the HubCox and EN models associated with the hallmarks of cancer, given by the CHAT. (**a**) HubCox; (**b**) EN. The value corresponds to the number of hits found in the literature, where light and dark blue correspond to a low and high number of hits, respectively.

**Figure 8:**
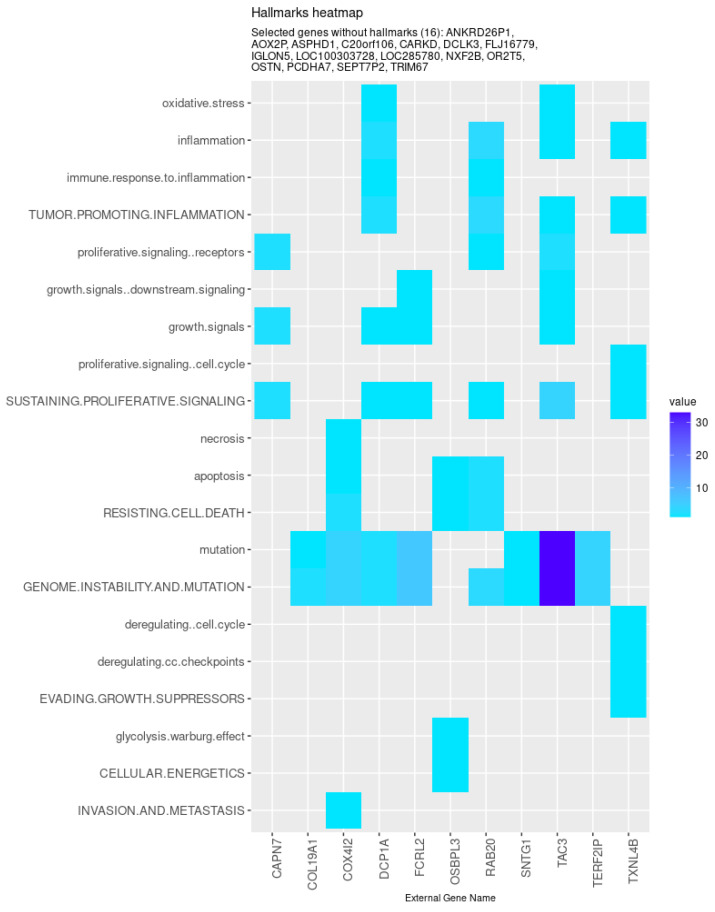
Genes selected by the TCox method associated with the hallmarks of cancer, given by the CHAT. The value corresponds to the number of hits found in the literature, where light and dark blue correspond to a low and high number of hits, respectively.

**Figure 9:**
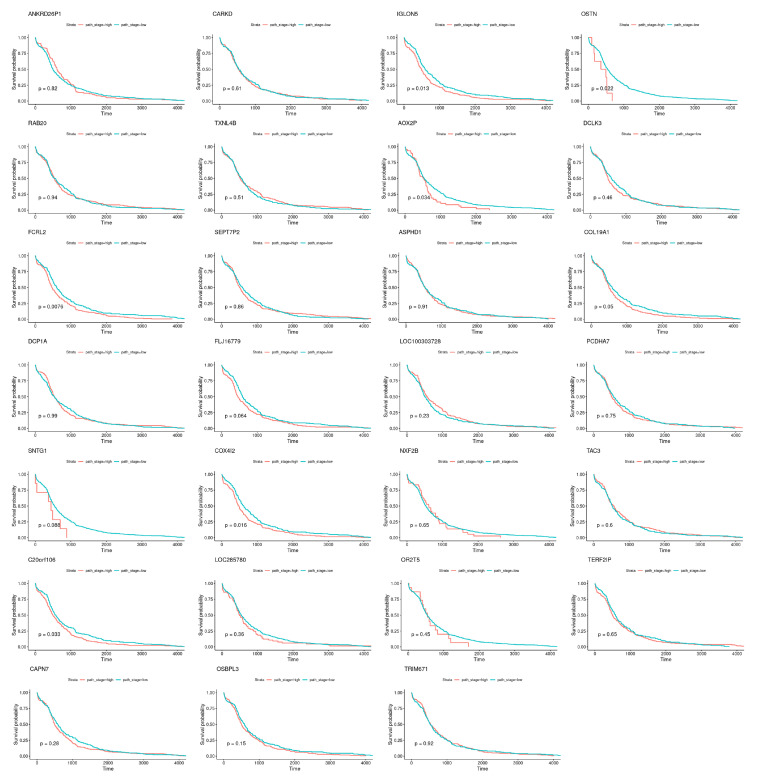
Survival curves obtained for the genes exclusively selected by the TCox method, when analyzed individually.

Nevertheless, specific genes were selected only by HubCox (8 genes), EN (6 genes), and TCox (27 genes), most of them with associations with the cancer hallmarks (Figure 7 and Figure 8). TCox was the model that identified the highest number of genes (Table 4); among them, eleven genes were associated with the hallmarks of cancer. In particular, the *RAB20*, *FCRL2*, *COL12A1*, *DCP1A*, and *OSBPL3* genes were previously shown to have prognostic value in cancer. In addition, pseudogenes (such as *ANKRD26P1*, *AOX2P*, and *SEPTIN7P2*) and genes involved in the integrity of the extracellular matrix (*COL19A1*), cellular adhesion (*IGLON5*, *PCDHA7*), the mitochondrial respiratory chain (*COX4I2*), telomere function (*TERF2IP*), E3 ubiquitination (*TRIM67*), and the export of nuclear RNA (*NXF2B*) suggested important roles in CRC development that should be further investigated. After analyzing each gene independently, we observed that most of the genes were not significantly associated with survival (Figure 9).

Finally, it is noteworthy that all the novel regularizers—either those favoring or penalizing the selection of hubs (HubCox and OrphanCox) or promoting the genes with distinct correlation patterns in tumor and normal tissue samples (TCox)—added valuable information to the results obtained by the Elastic Net only. Indeed, by significantly expanding the resulting gene sets, TCox generated hypotheses regarding putative targets that may be further tested and experimentally analyzed.

In the present study, we exclusively used RNA-seq data from TCGA. The inclusion of other clinical parameters is expected to improve the performance of the models. For example, the recent classification of CRC tumor subtypes (Consensus Molecular Subtypes (CMS1-4)) [42] may in the future contribute to a better set of biomarkers with higher prognostic value.

## 4. Conclusions

We propose TCox, a new weighted regularizer for Cox regression that penalizes the similarity of gene correlations across tumor and normal tissue samples in the selection of gene signatures associated with the survival outcome of colorectal cancer patients. Comparable model performance was obtained for TCox with respect to previously described methods in the literature, namely Elastic Net (EN), HubCox, and OrphanCox. Besides a consensus list of genes selected by all the regression models tested, with many of them already described to be involved in cancer formation and progression, TCox exclusively selected genes with an established role in colorectal cancer (CRC) and carcinogenesis, being able to categorize patients into significant risk groups. Regularized regression and, in particular, correlation-based Cox models are promising strategies to cope with high-dimensional data derived from multi-omics patient studies and can be useful to identify novel biomarkers in cancer.

## Figures and Tables

**Table 1:** Results from 100 runs of training and test sets in all survival models analyzed using α=0.1. S—statistically significant runs (*p*-value <0.05); NS—non-statistically significant runs; #—number of runs.

Models	TCox			EN			HubCox			OrphanCox		
**Runs** **Test set**	NA	S	NS	NA	S	NS	NA	S	NS	NA	S	NS
**#**	33	7	60	31	4	65	43	3	54	32	2	66
**Mean** ***p*** **-value**	–	0.0164	0.4985	–	0.0251	0.5354	–	0.0137	0.5168	–	0.0160	0.4997

**Table 2:** Summary of TCox, EN, HubCox, and OrphanCox model results showing the number of selected variables and the *p*-values obtained for the training and test sets.

Survival Models	α	Selected Variables	*p*-Value	
			Train	Test
**TCox** ($\frac{1}{w}$)	0.3	10	0.002401583	0.0757
	0.2	11	0.000588251	0.0665
	0.1	53	2.66444 × 10^−9^	0.0194
**EN**	0.3	18	8.38703 × 10^−7^	0.0088
	0.2	47	2.47428 × 10^−8^	0.0717
	0.1	88	5.28787 × 10^−9^	0.0492
**HubCox**	0.3	26	1.78804 × 10^−8^	0.0138
	0.2	47	1.18224 × 10^−8^	0.0129
	0.1	90	2.74104 × 10^−9^	0.0418
**OrphanCox**	0.3	8	2.48965 × 10^−5^	0.1519
	0.2	44	1.20494 × 10^−7^	0.0327
	0.1	67	6.80248 × 10^−9^	0.0632

**Table 3:** List of genes selected for at least 50% or 75% of the runs by all methods tested.

Runs		TCox	EN	HubCox	OrphanCox
**75%**	**#**	3	2	2	1
	**genes**	*GABRD, NKAIN4, ZIC3*	*ELFN1, LOC646498*	*ELFN1, LOC646498*	*LOC646498*
**50%**	**#**	16	16	16	1
	**genes**	*ASB10, ASPHD1, CST2,* *CT45A3, CYP19A1, DAD1L,* *ELFN1, FOXS1, GABRD, GH2,* *HIST1H2BG, HIST1H4H, NKAIN4,* *RHOXF2B, ZIC3, ZNF676*	*CLEC18C, EEPD1, ELFN1,* *HIST2H2BA, HIST2H2BE, KCNMB3,* *LOC100270710, LOC220930, LOC646498,* *NELF, ONECUT1, PRRX2,* *PRSSL1, RFPL4B, SIX2, TAS2R20*	*EEPD1, ELFN1, HIST1H2AE,* *HIST2H2BA, HIST2H2BE, KCNMB3,* *LOC100270710, LOC220930, LOC338758,* *LOC646498, NELF, ONECUT1,* *PRRX2, PRSSL1, TAS2R20, ZNF676*	*LOC646498*

**Table 4:** Genes selected by all models evaluated and selected exclusively by EN, HubCox, OrphanCox, and TCox. Arrows indicate if genes were found to be up- (↑) or down-regulated (↓) in tumoral tissue (differential gene expression analysis was performed using the edgeR R package).

**All models**	*CYP7A1 (↓), FAM159A (↓), ZNF883, CLDN9 (↑), LBX2 (↑), MEIG1, PAX5 (↓),*
	*NKAIN4 (↓), ZDHHC19 (↓), GRAPL, PCDHB12 (↓), EEPD1 (↑), HPCAL1,*
	*PGAM2 (↓), LOC732275, FAM138B (↓), LOC646498, PRCD (↓)*
**EN**	*HOTAIR (↑), GJA3 (↑), LOC283663 (↓), DNAI2 (↓), NELF (↑), GUCA1B*
**HubCox**	*CYGB (↓), UNC13B, LIPT2 (↑), RFT1 (↑), BEND4 (↓), FAM24B (↑), SLFN11, RASGRP2 (↓)*
**TCox**	*ANKRD26P1 (↑), CARKD, IGLON5, OSTN (↓), RAB20, TXNL4B (↑), AOX2P,*
	*DCLK3 (↑), FCRL2 (↓), SEPT7P2 (↑), ASPHD1 (↑), COL19A1 (↓), DCP1A,*
	*FLJ16779 (↑), LOC100303728 (↓), PCDHA7, SNTG1, COX4I2, NXF2B (↑),*
	*TAC3 (↓), C20orf106, LOC285780 (↓), OR2T5, TERF2IP, CAPN7, OSBPL3 (↑), TRIM67 (↓)*

## Abbreviations

The following abbreviations are used in this manuscript:

CHAT	Cancer Hallmarks Analytics Tool
COAD	Colon Adenocarcinoma
CRC	Colorectal Cancer
EN	Elastic Net
FPKM	Fragments Per Kilobase per Million
GDC	Genomic Data Commons
READ	Rectum Adenocarcinoma
RNA-seq	RNA sequencing
TCGA	The Cancer Genome Atlas
